# Angiotensin II and the Renal Hemodynamic Response to an Isolated Increased Renal Venous Pressure in Rats

**DOI:** 10.3389/fphys.2021.753355

**Published:** 2021-11-16

**Authors:** Xiaohua Huang, Shereen M. Hamza, Wenqing Zhuang, William A. Cupples, Branko Braam

**Affiliations:** ^1^Division of Nephrology, Department of Medicine, University of Alberta, Edmonton, AB, Canada; ^2^The First Affiliated Hospital of Shantou University Medical College, Shantou, China; ^3^Department of Physiology, University of Alberta, Edmonton, AB, Canada; ^4^Department of Biomedical Physiology and Kinesiology, Simon Fraser University, Burnaby, BC, Canada

**Keywords:** renal venous pressure, cardiorenal syndrome, renin – angiotensin – aldosterone system, renal hemodynamics, venous congestion

## Abstract

Elevated central venous pressure increases renal venous pressure (RVP) which can affect kidney function. We previously demonstrated that increased RVP reduces renal blood flow (RBF), glomerular filtration rate (GFR), and renal vascular conductance (RVC). We now investigate whether the RAS and RBF autoregulation are involved in the renal hemodynamic response to increased RVP. Angiotensin II (ANG II) levels were clamped by infusion of ANG II after administration of an angiotensin-converting enzyme (ACE) inhibitor in male Lewis rats. This did not prevent the decrease in ipsilateral RBF (−1.9±0.4ml/min, *p*<0.05) and GFR (−0.77±0.18ml/min, *p*<0.05) upon increased RVP; however, it prevented the reduction in RVC entirely. Systemically, the RVP-induced decline in mean arterial pressure (MAP) was more pronounced in ANG II clamped animals vs. controls (−22.4±4.1 vs. −9.9±2.3mmHg, *p*<0.05), whereas the decrease in heart rate (HR) was less (−5±6bpm vs. −23±4bpm, *p*<0.05). In animals given vasopressin to maintain a comparable MAP after ACE inhibition (ACEi), increased RVP did not impact MAP and HR. RVC also did not change (0.018±0.008ml/minˑmmHg), and the reduction of GFR was no longer significant (−0.54±0.15ml/min). Furthermore, RBF autoregulation remained intact and was reset to a lower level when RVP was increased. In conclusion, RVP-induced renal vasoconstriction is attenuated when ANG II is clamped or inhibited. The systemic effect of increased RVP, a decrease in HR related to a mild decrease in blood pressure, is attenuated also during ANG II clamp. Last, RBF autoregulation remains intact when RVP is elevated and is reduced to lower levels of RBF. This suggests that in venous congestion, the intact RBF autoregulation could be partially responsible for the vasoconstriction.

## Introduction

Increased renal venous pressure (RVP) is associated with worsening of renal function in congestive states, particularly heart failure (Damman et al., 2009; Mullens et al., 2009). Nevertheless, the mechanisms mediating the negative impact of increased RVP on renal function are incompletely understood. In a previous study, we demonstrated that an acute, isolated increase of RVP leads to reduction of renal vascular conductance (RVC; Huang et al., 2018). Interestingly, the RVC reduction was strongly attenuated in rats receiving a high salt diet but was unchanged after renal denervation. This suggested that the renin-angiotensin system (RAS) contributes to the renal vasoconstriction upon increased RVP. Indeed, Kastner et al. (1982) showed renin levels were profoundly increased due to RVP elevation. However, in their study, an increase in RVP after RAS inhibition was associated with a more profound decrease in glomerular filtration rate (GFR). Therefore, the role of the RAS in the renal hemodynamic and systemic response to RVP elevation remains unclear. Understanding this is relevant, since RAS inhibitors are frequently used in patients with heart failure and the (dis)continuation of these drugs in the context of worsening of renal function in heart failure is highly disputed.

The RAS could affect the renal hemodynamic response to RVP directly by causing vasoconstriction but also indirectly by enhancing renal blood flow (RBF) autoregulation (Sorensen et al., 2000; Cupples and Braam, 2007). Since an increase in RVP could cause an increase in afferent arteriolar pressure, it could provoke an autoregulation-mediated afferent arteriolar vasoconstriction. If autoregulation is enhanced due to increased RAS activity upon increased RVP, RBF, and GFR could become depressed.

Therefore, one aim of the present study was to test whether the vasoconstriction caused by an isolated increase of RVP is due to activation of the RAS. We measured the hemodynamic response to increased RVP after inhibition of endogenous angiotensin II (ANG II) formation using angiotensin-converting enzyme inhibition (ACEi) followed by exogenous “clamp” of circulating ANG II. We also studied the situation where ANG II is absent, by complete blockade with an ACEi and restoration of arterial pressure using vasopressin infusion (AVP). The second aim was to investigate the response of overall RBF autoregulation to isolated increases in RVP, which was performed by stepwise reduction in renal perfusion pressure (RPP).

## Materials and Methods

All experiments were conducted in accordance with the guidelines of the Canadian Council on Animal Care and received prior approval by the Animal Care and Use Committee of the University of Alberta. Male Lewis rats (350–450g; Charles River, St. Constant, QC, Canada) were housed in a temperature-controlled (22–26°C) and humidity-controlled (60–70%) room with a 12-h:12-h light/dark cycle. All rats (*n*=55) received regular rat chow containing 1% NaCl *ad libitum* (Canadian Lab Diets, Leduc, AB, Canada) and had free access to tap water.

### General Animal Preparation

Rats were given buprenorphine (0.02mg/kg, i.m.) 30min before anesthesia. Animals were placed in an induction chamber pre-charged with room air, and isoflurane was introduced in 0.5% increments up to 4% in 100% oxygen (1L/min). Once the rat reached surgical anesthetic plane, it was placed on a heated surgical table equipped with a thermo-feedback system to maintain body temperature between 36 and 37°C (Vestavia Scientific, Birmingham, AL, United States). Anesthesia was maintained through a nose cone, and the isoflurane dose was gradually reduced in 0.5% increments to 2%. Rats were breathing spontaneously. The neck, abdomen, and left groin were shaved, and the surgical field was cleaned with alternating applications of 10% povidone iodine and 70% ethanol. Following a midline neck incision to expose the trachea, the rat was intubated *via* tracheotomy using PE-240 tubing (BD Intramedic, Sparks, MD, United States). The tracheal tube was then fitted to the nose cone, and isoflurane dose was adjusted to 1.5–1.75% to maintain surgical plane with the loss of the toe pinch reflex.

The left femoral vein was catheterized (Silastic tubing, 0.51mm ID, 0.94mm OD, Dow Corning, Midland, MI, United States) for intravenous infusion, which was immediately commenced (see below). The left femoral artery was cannulated (PE-50, BD Intramedic, Sparks, MD, United States) for direct recording of systemic arterial pressure and heart rate (HR). Following midline laparotomy, the left kidney was exposed. The left adrenal vein or supraspermatic vein was cannulated (Micro-Renathane MRE-025, Braintree Scientific, Braintree, MA, United States) and the cannula advanced until the tip rested in the main renal vein for direct measurement of RVP. A length of 3–0 prolene (Johnson-Johnson, San Lorenzo, Puerto Rico) was slipped around the left renal vein at its junction with the inferior vena cava and sheathed with a small piece of PE-50 tubing to create a sling. To increase RVP, the sling was tightened to partially constrict the renal vein. Pressures were acquired using PowerLab *via* disposable blood pressure transducers (ADInstruments, CO, United States). A 1RB transit-time flow probe was placed around the left renal artery for direct measurement of RBF (Transonic, Ithaca, NY, United States). The left ureter was catheterized for gravimetric urine collection (PE-10, BD Intramedic, Sparks, MD, United States). The rat received supplemental fluids during surgical preparation (5% bovine serum albumin in normal saline, BSA, A7906, Sigma, Oakville, ON, Canada) with 250μg/min FITC-inulin (Sigma, Oakville, ON, Canada) at 1.5ml/h. This infusion continued throughout the experiment with 1% BSA with 250μg/min FITC inulin at 1.5ml/h.

### Experiment 1: ANG II Clamp Experiment

Enalapril was administered i.v. in a bolus of 0.2mg/kg BW and infused at 3μg/min in 18 rats (Turkstra et al., 2000b). Following completion of surgical instrumentation, angiotensin I (ANG I) was administered i.v. in a bolus of 25pmol to test the adequacy of ACE inhibition. ANG II (started at 0.25μg/kg/min) was continuously infused to restore mean arterial pressure (MAP) to baseline values. Rats were stabilized for 60min. Baseline data were collected for 60min after which RVP was selectively increased to 20mmHg (*n*=10) by partial constriction of the left renal vein or not manipulated (Time Control, TC, *n*=8).

### Experiment 2: ANG II Absent Experiment

This experiment was designed to demonstrate the hemodynamic responses to increased RVP in the absence of ANG II in a separate group of rats (*n*=15). Efficacy of ACE inhibition was tested as described above. AVP (started at 8ng/kg/min, Wang et al., 1999) was continuously infused to restore MAP to baseline values. Rats were stabilized for 60min. Baseline data were collected for 60min after which RVP was selectively increased to 20mmHg (*n*=8) by partial constriction of the left renal vein or not manipulated (Time Control, TC, *n*=7).

### Experiment 3: RBF Autoregulation Experiment

Renal blood flow autoregulation in response to RVP elevation was evaluated in a separate group of rats (*n*=22); surgical preparation was similar. In addition, a second sling was placed around the aorta above the left renal artery using a length of 3–0 prolene (Johnson-Johnson, San Lorenzo, Puerto Rico) sheathed with a small piece of PE-50 tubing. RBF measurements and infusions were as described above. Once instrumented and after a 60min equilibration, RPP was decreased by stepwise 10mmHg reduction of renal arterial pressure *via* partial occlusion of sling around the aorta as described previously (Turkstra et al., 2000a). Each step of the decrease was recorded for 5min, then the sling around the aorta was released, and the RBF was allowed to return to baseline for 5min. The maximum decrease was −40mmHg. The stepwise decrease was repeated when RVP was increased to either 10mmHg (RVP10, *n*=8), or 20mmHg (RVP20, *n*=8), or left at baseline (TC, *n*=6).

### Measurement of GFR

Baseline data were collected for 60min, after which time RVP was selectively increased to 20mmHg by graded constriction of the left renal vein or not manipulated (TC). Data collection continued for a further 120min. For hemodynamic experiments, blood samples (200μl) were obtained at the beginning of the baseline period and every 30min thereafter. Timed urine samples were collected every 30min. No blood or urine sampling was done in the autoregulation experiments. To determine GFR using FITC-Inulin, plasma and urine samples were diluted in 0.5mol/L HEPES (pH 7.4) to maintain physiological pH. A 96-well black plate (Greiner, Monroe, NC, United States) was used for loading 50μl of each solution in duplicate. Fluorescence was determined using the Fluoroskan Ascent® Microplate Fluorometer (Thermo Fisher Scientific, Vantaa, Finland), at the excitation wavelength of 485nm and emission wavelength of 527nm.

### Analysis and Statistics

Hemodynamic data are presented as the average of consecutive 30min intervals. The baseline characterization was compared between both ANG II Clamp and ANG II Absent rats using a general linear model multivariate ANOVA (MANOVA) with Bonferroni *post hoc* test. To evaluate the impact of elevated RVP, a multiple linear model with repeated measurement was used to compare each time point of different RVP groups, using Bonferroni as *post hoc* test. Data were log-transformed or ranked if not normally distributed. Data were analyzed using SPSS 24 (IBM, Armonk, NY, United States).

To evaluate RBF autoregulation, a baseline of RPP and RBF was obtained before any constriction of the aorta. For each step of RPP reduction, the RBF was allowed to stabilize for 1min and the average value of the subsequent 4min was used to calculate the autoregulation curves. The autoregulation curves were plotted and analyzed in SigmaPlot 13 (Systat, San Jose, CA, United States) using nonlinear regression analysis. The lower limit of RBF autoregulation was defined as the perfusion pressure at which the third derivative of the fitted curve was 0 (Turkstra et al., 2000a). The lower limit of RPP for autoregulation was compared using two-way ANOVA with Student-Newman-Keuls *post hoc* test. Statistical significance was accepted at *p*<0.05. All data are presented as means±SEM.

## Results

### Increased RVP and Hemodynamics in ANG II Clamp and ANG II Absent Rats

**Figure 1:**
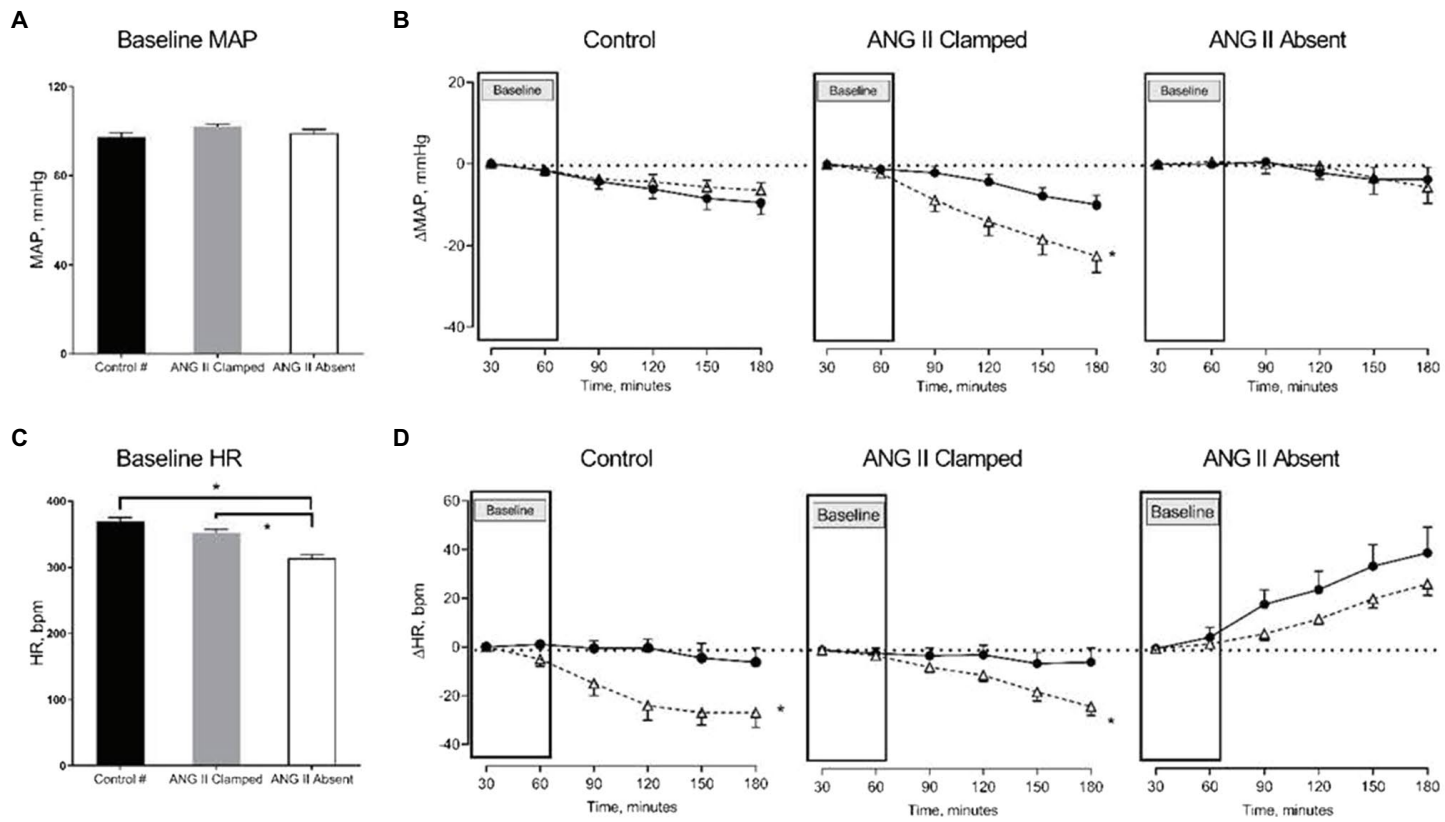
Changes in mean arterial pressure (MAP) and heart rate (HR) in response to increased renal venous pressure (RVP). The bar graphs show the baseline MAP **(A)** and HR **(C)** in untreated control (black, control, *n*=8), angiotensin II (ANG II) Clamp (grey, ANG II Clamp, *n*=10) and ANG II Absent (open bar, ANG II Absent, *n*=8, BP supported by AVP) rats. The line charts represent changes from baseline, △MAP **(B)**, and △HR **(D)**. In the line chart, the first two time points in each line represent baseline. The latter four time points show control (●, control) and major RVP elevation (△, RVP20). Baseline MAP did not differ among groups **(A)**. Increased RVP caused a significant decrease in MAP in the ANG II Clamp group (**B**; ^*^p<0.05) but not in untreated control group or ANG II Absent group. Baseline HR was significantly lower in the ANG II Absent group than in untreated control and ANG II Clamp groups (**C**; ^*^p<0.05). Increased RVP induced HR reduction in untreated control group and ANG II Clamp group (^*^p<0.05) but not in the ANG II Absent group **(D)**. #Data from these were published in Huang et al. (2018).

Baseline characteristics are presented in Table 1. There were no significant differences in BW, HCT, and baseline MAP between the ANG II Clamp and ANG II Absent groups. In the absence of ANG II, the baseline RBF, GFR, and RVC were higher than in the ANG II Clamp group. Baseline HR was lower in the ANG II Absent rats than in controls or ANG II Clamp groups, shown in Figure 1C.

**Table 1:** Baseline information in untreated control, ANG II Clamp, and ANG II Absent rats.

	Untreated controls	ANG II clamp	ANG II absent
*n*	17	18	15
BW, g	373±8	395±11	382±7
HCT, %	43.8±0.5	46.2±0.7	45.5±0.4
MAP, mmHg	97±2	102±1	99±2
HR, bpm	370±5	352±5	313±6^*^^‡^
RBF, ml/min	7.2±0.6	6.1±0.5	10.2±0.8^*^^‡^
RVP, mmHg	0.4±0.1	0.2±0.2	0.2±0.2
RVC, ml/minˑmmHg	0.075±0.006	0.060±0.006	0.103±0.008^*^^‡^
GFR, ml/min	1.42±0.07	1.37±0.08	1.64±0.06^*^^‡^

* *p*<0.05 vs. untreated control.

‡ *p*<0.05 vs. ANG II clamp.

### Systemic Hemodynamic Effects in ANG II Clamp and ANG II Absent Groups Upon Increased RVP

**Figure 2:**
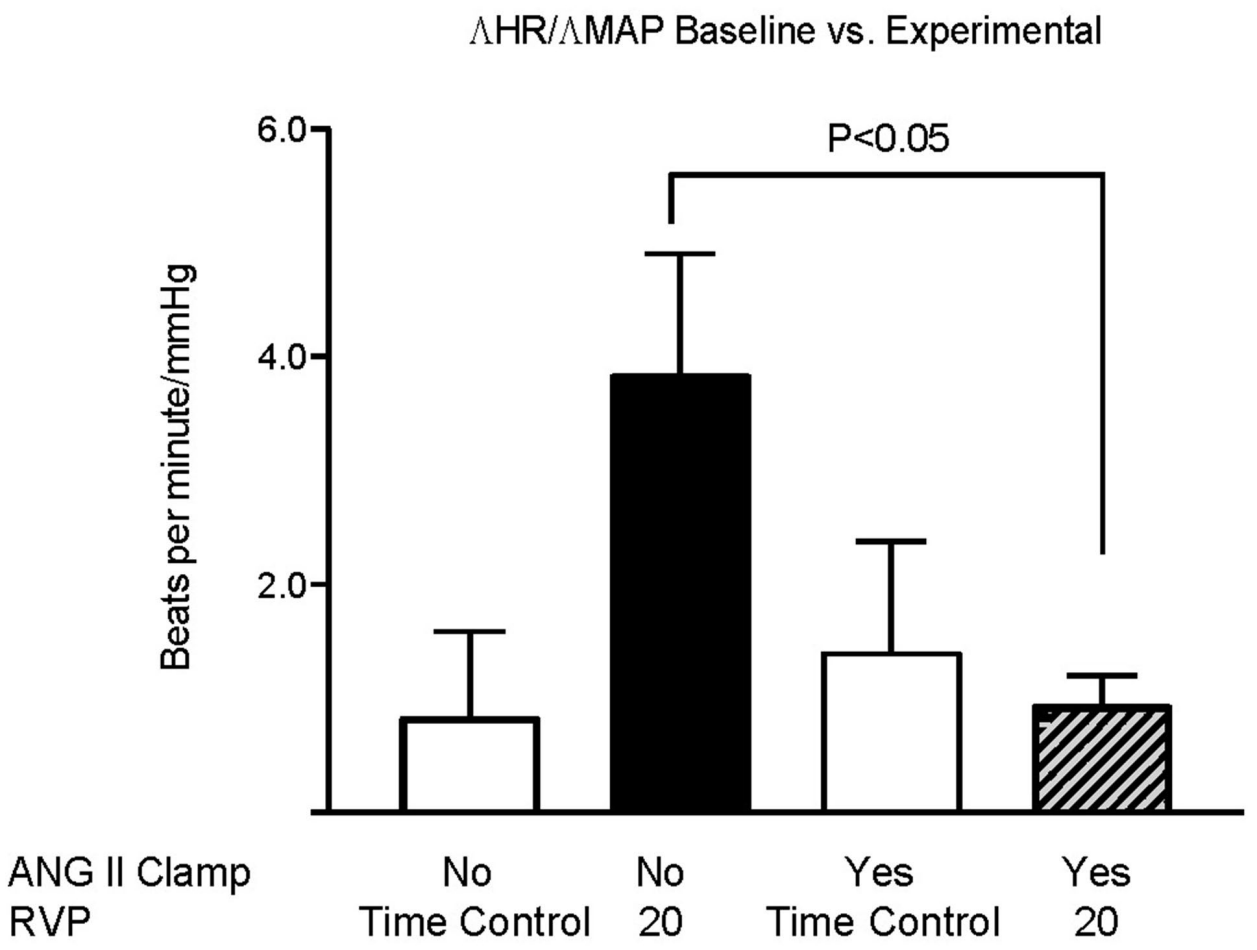
Changes in HR per changes in MAP in untreated rats and ANG II Clamp rats. The ratio of the change in heart rate vs. the change in MAP of the baseline measurements vs. the measurements at 90 and 120min was calculated for each animal. Groups, untreated Time Controls (TC, *n*=9), untreated animals subjected to increased RVP (*n*=8), ANG II Clamp time controls (not subjected to an increase in RVP, *n*=8), and ANG II Clamp animals subjected to increased RVP (*n*=10) were compared using two-way ANOVA with Bonferroni correction.

Baseline MAP did not differ among the three groups (Figure 1A). We had previously observed that an increase in RVP to 20mmHg did not significantly affect MAP, but decreased HR (this control is included in Figures 1B,D; Huang et al. 2018). In ANG II Clamp animals, an acute increase of RVP to 20mmHg induced a gradual reduction in MAP from 102±1 to 78±5mmHg (*p*<0.05; Figure 1B) and also a decrease in HR from 359±5 to 337±8bpm (*p*<0.05; Figure 1D). Further analysis of the change in MAP vs. heart rate is depicted in Figure 2. In the control animals, a relatively small decrease in MAP upon an increase in RVP to 20mmHg was accompanied by a strong decrease in HR, which was not observed in the time control animals with or without ANG II Clamp. In the ANG II Clamp animals where RVP was increased to 20mmHg, the decreases in MAP and HR were blunted (Figure 2).

### Renal Hemodynamic Effects in ANG II Clamp and ANG II Absent Groups Upon Increased RVP

**Figure 3:**
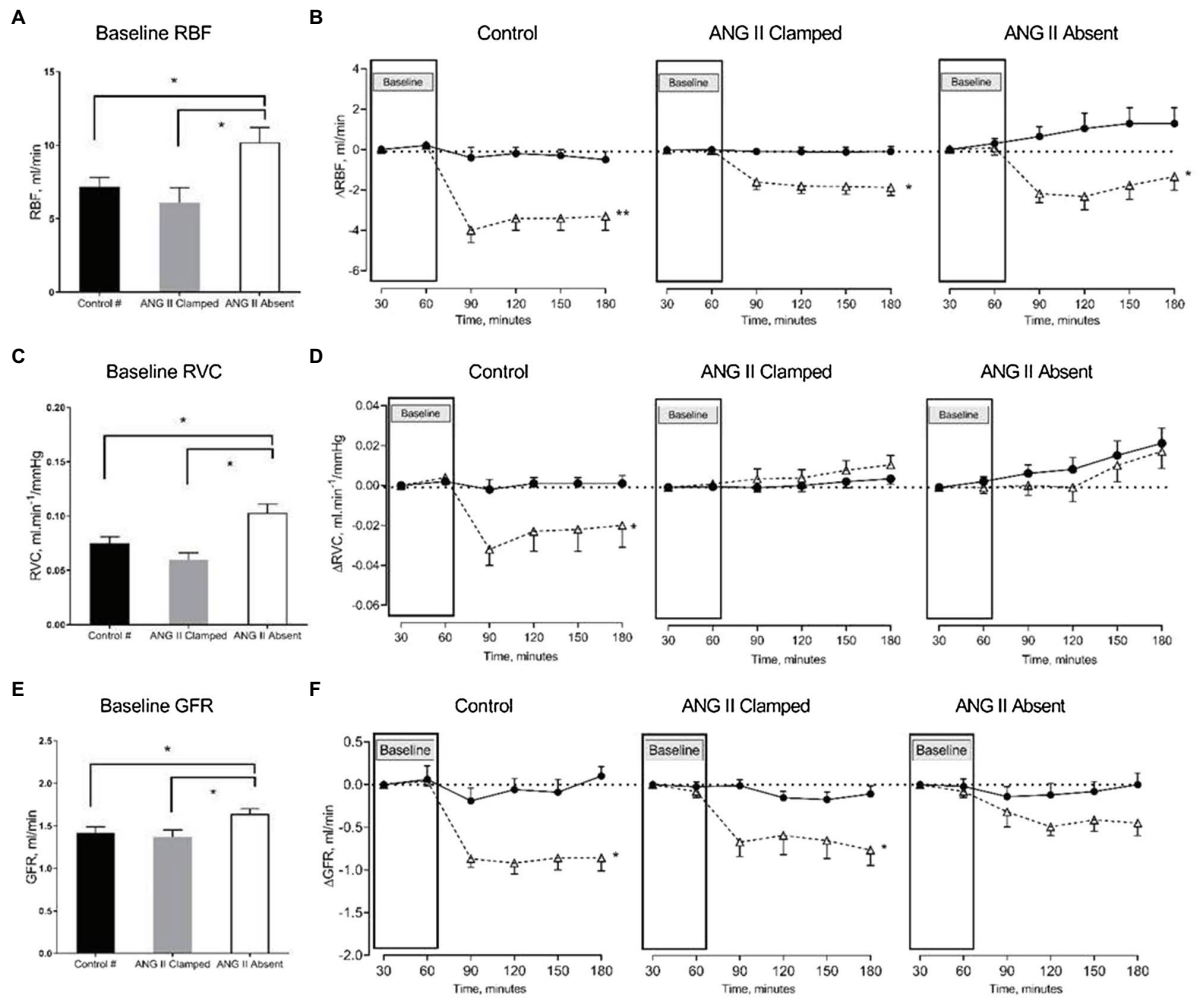
Changes in renal blood flow (RBF), renal vascular conductance (RVC), and glomerular filtration rate (GFR) in response to increased RVP. The bar graphs show the baseline RBF **(A)**, RVC **(B)**, and GFR **(C)** in untreated control (black, control, *n*=8), ANG II Clamp (gray, ANG II Clamp, *n*=10) and ANG II Absent (open bar, ANG II Absent, *n*=8, BP supported by AVP) rats. The line charts show changes from baseline, △RBF **(B)**, △RVC **(D)**, and △GFR **(F)**. In the line chart, the first two time points in each line represent baseline. The latter four time points show control (●, control) and major RVP elevation (△, RVP20). Baseline RBF was significantly higher in the ANG II Absent group than in untreated control and ANG II Clamp group (**A**; ^*^p<0.05). Elevation of RVP reduced RBF in untreated control group (**B**; ^**^p<0.001), in ANG II Clamp and ANG II Absent groups (**B**; ^*^p<0.05). Baseline RVC was higher in ANG II Absent group (**C**; ^*^p<0.05). Increased RVP reduced RVC in untreated control group (**D**; ^*^p<0.05), but did not impact RVC in either ANG II Clamp or ANG II Absent group. Baseline GFR was higher in the ANG II Absent group than in the other groups (**E**; ^*^p<0.05). Increased RVP caused significant reduction of GFR in both untreated and ANG II Clamp rats (**F**; ^*^p<0.05), while the apparent decrease in ANG II Absent rats was not statistically significant (*p*=0.081). #Data from these were published in Huang et al. (2018).

In both groups, inhibition of RAS did not prevent the reduction of RBF in response to an increased RVP. RBF decreased when RVP was increased, and the reduction in RBF was sustained until the end of the experiment. Comparing baseline and the end of the experiment, RBF decreased from 6.3±0.8 to 4.5±0.8ml/min in the ANG II Clamp group (*p*<0.05) and from 10.8±1.2 to 9.6±1.2ml/min in the ANG II Absent group (*p*<0.05). Of note, the change in RBF occurred within 30min of increased RVP and then remained stable (in the ANG II Clamp group) or slightly recovered (in the controls and the ANG II Absent group). Other than in the untreated control animals, in both the ANG II Clamp and ANG II Absent groups, the increase in RVP was not associated with a decrease in RVC. However, the inhibition of RAS did not prevent the decrease of GFR. In the ANG II Clamp rats, GFR decreased from 1.34±0.09 to 0.61±0.14ml/min in response to RVP elevation (*p*<0.05). Although not statistically significant, GFR showed a tendency to decrease from 1.57±0.06 to 1.05±0.13ml/min (*p*=0.081) in the ANG II Absent group (Figure 3).

### Increased RVP and Autoregulation of RBF

**Figure 4:**
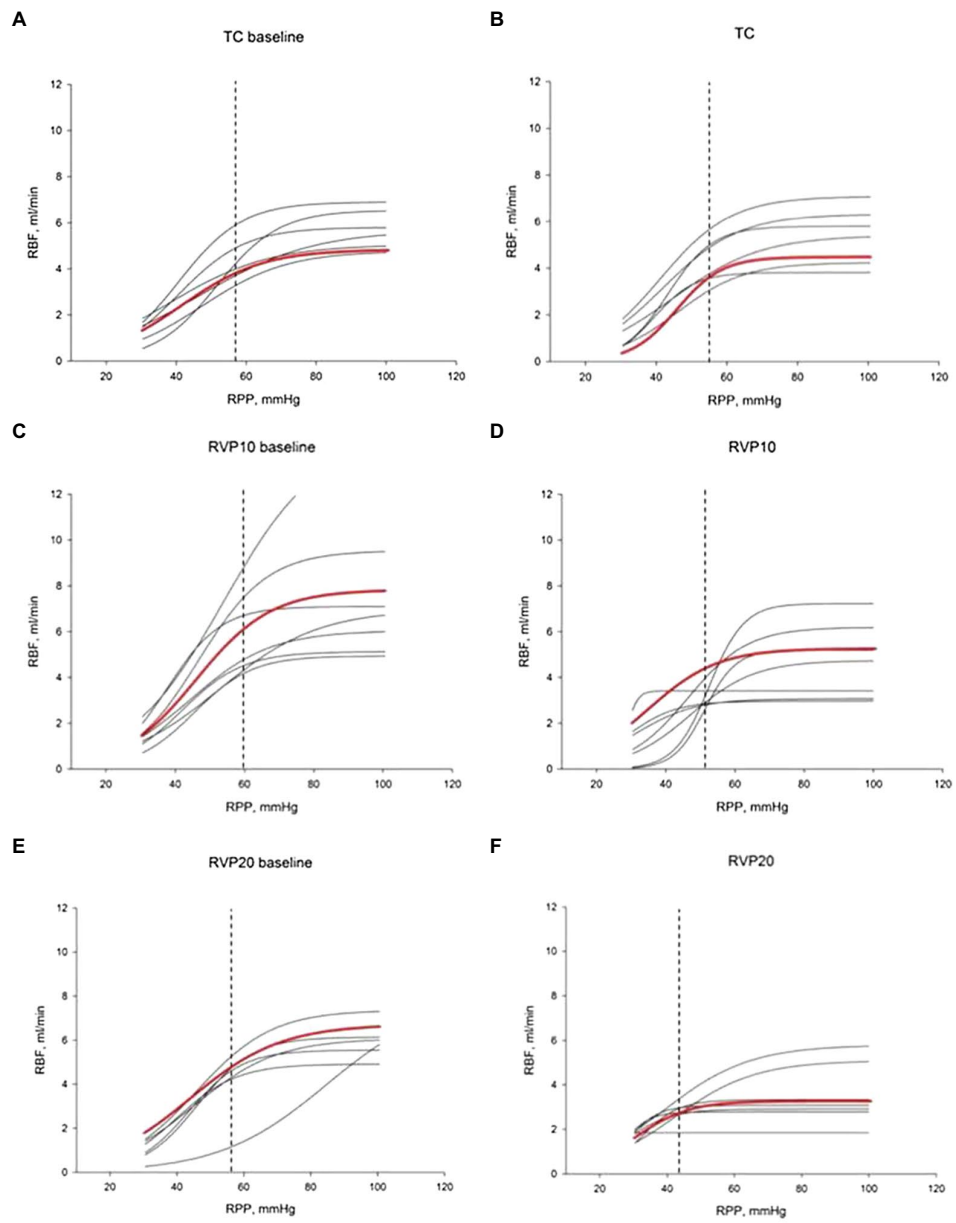
Renal blood flow autoregulation curves. Each curve is generated by nonlinear regression analysis. **(A,C,E)** show baseline autoregulation curves before RVP manipulation. **(B,D,F)** show autoregulation curves without RVP elevation (**B**, TC, *n*=6), with RVP elevation of ~10mmHg (**D**, RVP 10, *n*=8) and with RVP elevation of ~20mmHg (**F**, RVP 20, *n*=8). The red curve indicates the average of the curves. The lower limit of RBF autoregulation is indicated by the vertical dashed line.

The lower limit of RPP for the RBF autoregulation was calculated using a sigmoidal curve fit as we have published before (Turkstra et al., 2000a). There were no significant differences in baseline RVP, MAP, or RBF among the three groups. A moderate increase in RVP from 0.1±0.4 to 11.0±0.4mmHg resulted in a decrease of the lower limit from 62±3 to 52±3mmHg (*n*=8; *p*=0.053). A major increase of RVP from 0.7±0.2 to 19.1±0.4mmHg in separate experiments caused a significant decrease of the lower limit from 57±2 to 43.6±4.3mmHg (*n*=8; *p*<0.05; Figure 4).

## Discussion

In our previous study, a substantial increase in RVP by 20mmHg caused a significant reduction in RBF, RVC, and GFR (Huang et al., 2018). In the current study, the RVP-induced reduction in RVC was prevented in the absence of dynamic ANG II modulation (ANG II Clamp) and in the absence of ANG II (AVP infusion with ACEi). This suggests that RVP-induced vasoconstriction is not dependent on the presence of ANG II *per se*, but on modulation of ANG II. Furthermore, we demonstrated that autoregulation of RBF remained intact when RVP was elevated, yet the lower limit of autoregulation decreased progressively to a lower level of perfusion pressure with increasing RVP.

Our findings are consistent with the findings from others that RVP elevation activates the RAS. In a report in 1972, increased RVP resulted in increased renin secretion in dogs (Schmid, 1972). Kishimoto et al. (1973) also showed in dogs that increased RVP (30mmHg) increased renin secretion, and it was suggested this might be triggered by a shift in blood flow from the outer to inner cortex. Similarly, Kopp et al. (1984) showed that raising RVP to 28mmHg increased ipsilateral renin secretion in dogs, which was related to the reno-renal reflex. A similar finding in dogs that elevated RVP (30mmHg) increased renin secretion especially when RVP was greater than 0mmHg and to rates 16-fold above baseline when RVP was 50mmHg (Kastner et al., 1982). Remarkably, in their study, GFR remained stable over a range of RVP elevation from 0 to 50 mmHg. Furthermore, in their study, inhibition of the RAS caused a greater reduction in GFR in response to increased RVP, which also contrasts with our findings. The opposite responses might be because the lack of the clamped level of ANG II as in our study or perhaps indicating species-specific effects since their study employed dogs. In our study, when the ANG II level was fixed or completely absent, RVP-induced reduction of RBF, RVC, and GFR was attenuated. Taken together, our current and previous findings indicate an important functional role for increased activity of the RAS when RVP is elevated.

Increased RVP affects systemic hemodynamic regulation. In our previous study, an increase in RVP to 20mmHg was associated with a mild decrease in MAP and a clear decrease in HR. This indicates that RVP dampens the arterial baroreceptor reflex. In the ANG II Clamp rats, however, this inhibitory effect on the baroreflex was attenuated. These observations raise several questions. First, could the renal nerves be involved? Endogenous ANG II can impair the renal mechanosensory nerves therefore suppressing the afferent renal nerve activity (Kopp et al., 2003, 2007). In our previous study, we demonstrated that the decrease in HR also occurred when the kidneys were denervated. Admittedly, AVP could potentiate the baroreflex (Cowley et al., 1974; Goldsmith, 1997); however, in our ANG II Clamp experiments, the depression of the baroreflex was also attenuated. ANG II has been shown to attenuate the sympathetic baroreflex function and reduce the baroreflex gain (HR/MAP; Guo and Abboud, 1984). Other data suggest that ANG II shifts the baroreflex to higher pressures without altering the sensitivity, contrary to the current findings (Ligtenberg et al., 1999). Could it also be possible that another factor is activated by ANG II? In this regard, a potential candidate that cannot be overlooked is nitric oxide. Nitric oxide has been shown to blunt baroreflex control of HR in rats (Abdulla and Johns, 2014). The present findings suggest that interactions among ANG II and other mediators determine the systemic impact of increased RVP.

**Figure 5:**
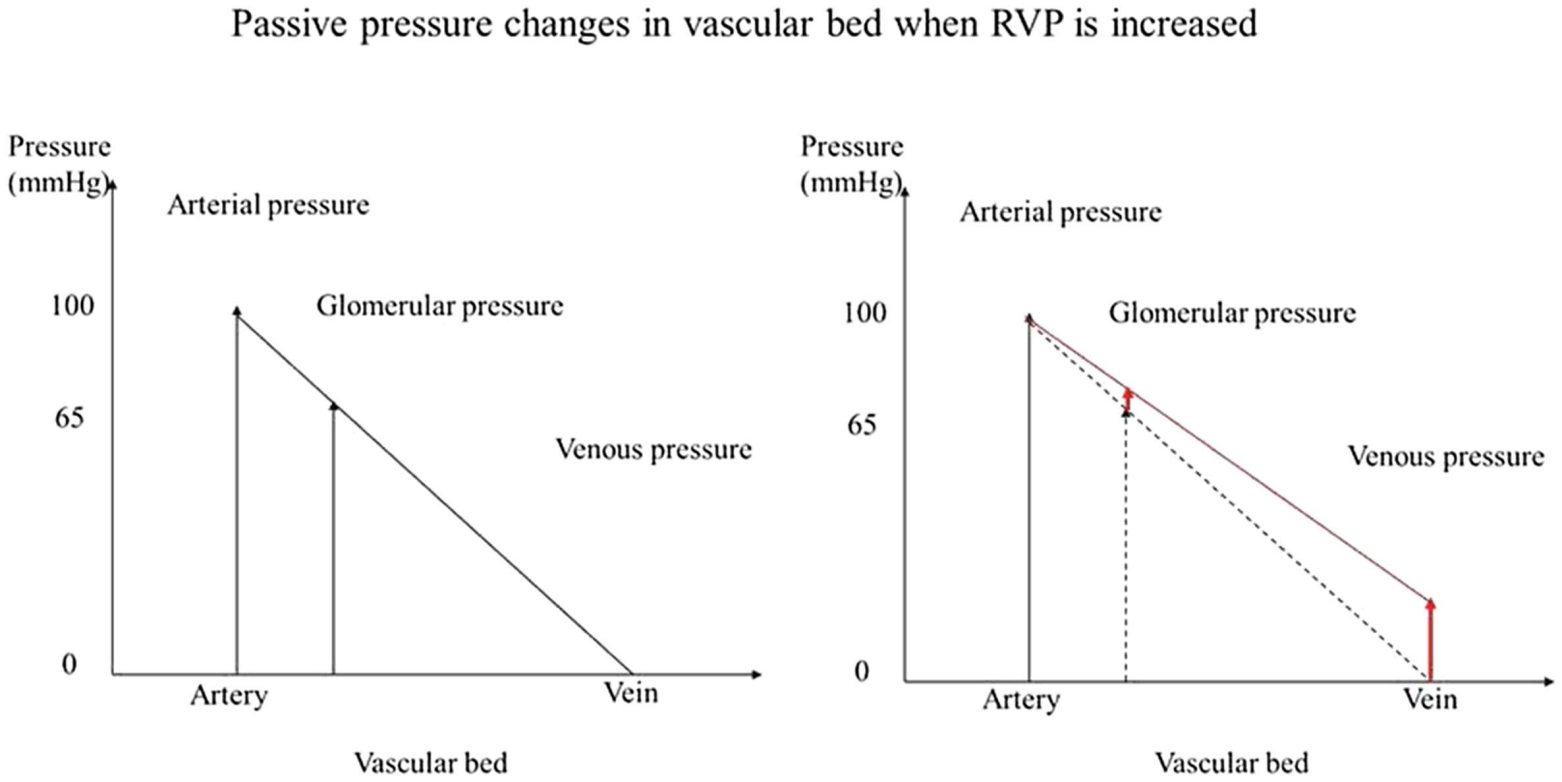
Passive pressure changes in vascular bed when RVP is increased. When pressure is increased in the venous system without neurohumoral adjustment, the glomerular capillary hydrostatic pressure is increased passively.

We also studied RBF autoregulation in response to RVP elevation, since increased RVP without neurohumoral adjustment will passively increase the glomerular capillary hydrostatic pressure (Figure 5). The elevation of preglomerular pressure could evoke an afferent arteriolar vasoconstriction. Alternatively, increased glomerular capillary hydrostatic pressure could increase GFR, increase distal delivery, and trigger vasoconstriction in the afferent arterioles *via* tubuloglomerular feedback. However, the increased RVP also increases the tubular pressure as well as interstitial pressure, which makes the overall autoregulatory response uncertain. Studies in rats and dogs have shown that tubuloglomerular feedback was unaffected by an increase in RVP to 20mmHg (Bell and Navar, 1979; Boberg and Persson, 1985). On the other hand, the other component of autoregulation, the myogenic response, has not been studied. It could also contribute to vasoconstriction due to the increased afferent arteriolar pressure when RVP is increased. Our finding that autoregulation of RBF is not impaired by the increased RVP indicates that autoregulation is another contributor to the RVP-induced vasoconstriction. While during acute venous congestion the kidney is able to maintain relatively stable RBF in response to further fluctuation in RPP, this comes at the cost of vasoconstriction. In chronic venous congestion, other mediators might become involved. For example, NO contributes to the vasoconstriction that occurs when MAP is reduced for at least 10min in spontaneously hypertensive rats (Sorensen et al., 2003). Our finding could imply that autoregulation participates in the acute vasoconstriction response to the RVP elevation.

Taken together, our previous and current findings are consistent with a primary role of the RAS in the renal hemodynamic responses to increased RVP. The renal vasoconstriction induced by elevated RVP was prevented by inhibition of ANG II. In addition, RBF and GFR were better preserved without dynamic modulation of ANG II or in the absence of ANG II than in the untreated animals. Increased RVP tends to blunt the baroreflex by the interaction between RAS and other mediators. The intact RBF autoregulation and decreased lower limit in renal venous congestion is important because it protects the kidney in case of a sudden arterial pressure insufficiency, but it also causes vasoconstriction. Further studies are needed in a chronic setting and in pathophysiological models.

### Perspectives

Our current study looked at two potential mechanisms behind RVP-induced vasoconstriction and at the impact of systemic hemodynamics. The role of RAS is of interest because pharmacological inhibition of the RAS is widely used in the treatment of congestive heart failure together with pharmacological decongestion. However, the impact of RAS inhibitors on renal hemodynamics and excretory function is not well-studied in combination with diuretics. Intact autoregulation is also important in maintaining GFR at the cost of vasoconstriction. These mechanisms are studied under an acute increase of RVP. It is commonly assumed that inappropriate activation of autoregulation, particularly of tubuloglomerular feedback, contributes to the impaired renal function seen in heart failure. However, there is currently little evidence to support that assumption. Further investigations are needed to study the endogenous adaptations of the kidney in chronic venous congestion as well as in disease model.

## Data Availability Statement

The raw data supporting the conclusions of this article will be made available by the authors, without undue reservation.

## Ethics Statement

The animal study was reviewed and approved by Animal Care and Use Committee of the University of Alberta.

## Author Contributions

BB and WC contributed to conception and design of the study. XH, SH, and WZ contributed to the acquisition of data. XH organized the database, performed the statistical analysis, and wrote the first draft of the manuscript. All authors contributed to the article and approved the submitted version.

## Funding

BB received a Grant-in-Aid from the Heart and Stroke Foundation of Canada and also holds the Kidney Health Translational Research Chair funded by the Division of Nephrology, Department of Medicine, Faculty of Medicine and Dentistry, University of Alberta. XH was supported by the Li Ka Shing Foundation.

## Conflict of Interest

The authors declare that the research was conducted in the absence of any commercial or financial relationships that could be construed as a potential conflict of interest.

## Publisher’s Note

All claims expressed in this article are solely those of the authors and do not necessarily represent those of their affiliated organizations, or those of the publisher, the editors and the reviewers. Any product that may be evaluated in this article, or claim that may be made by its manufacturer, is not guaranteed or endorsed by the publisher.
